# Parents and Peers in Child and Adolescent Development: Preface to the Special Issue on Additive, Multiplicative, and Transactional Mechanisms

**DOI:** 10.3390/children8100831

**Published:** 2021-09-22

**Authors:** Amanda W. Harrist, Michael M. Criss

**Affiliations:** Department of Human Development & Family Science, Oklahoma State University, Stillwater, OK 74078, USA; michael.criss@okstate.edu

## Abstract

Parents and peers play critical roles in the socialization of children and adolescents, yet investigations on the role played by parents vs. peers have been largely separate for many years. To address this problem, we invited leading scholars in the field to collectively tell a complex story of the part that parents and peers together play in the development of children and adolescents. The resulting Special Issue is a collection of papers highlighting current conceptualizations and empirical work in this area, with a focus on additive, multiplicative, and transactional mechanisms that link parent and peer relational contexts to each other and to child/adolescent social and emotional development. Two papers present new conceptual models, six illustrate empirical work in the field, and one paper that provides a comprehensive review of the literature. The stories that are conveyed in the issue are both innovative and complex.

## 1. Trends in Parent and Peer Influence Research

The papers in this Special Issue are illustrative of both methodological and theoretical trends in the field of parent/peer socialization. 

**Methodological trends**. The papers in this Special Issue illustrate current methodological practices, particularly regarding sample selection. One innovation is the inclusion of both mothers and fathers in five of the six empirical studies in this Special Issue, an approach that was not often taken in past parent–peer literature. Additionally, child and adolescent samples are represented in the empirical papers: Assari et al. [1] and Jespersen et al. [2] studied children; Cox et al. [3], Havewala et al. [4], Hu et al. [5], Lindsey [6], and Sigal et al.’s [7] samples comprised adolescents; and Gazelle and Cui’s [8] included both age groups. This is in line with our belief that peer groups are critical contexts for socialization in both childhood and adolescence. Finally, the samples included greater diversity than many studies in the past: Black and Latinx populations were well represented in the Sigal et al. [7], Lindsey [6], and Gazelle and Cui samples [8]; Hu et al.’s [5] sample was from mainland China; Cox et al.’s [3] theory was developed to specifically address the experience of immigrant families; and the long-term goal for Assari et al.’s [1] research is to understand the effects of structural racism. 

**Figure 1:**
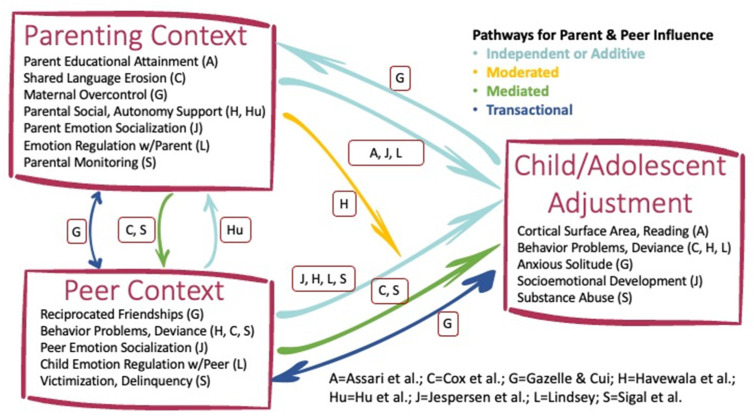
Pathways for parent and peer influences proposed in theoretical papers or supported empirically in this Special Issue.

As can be seen in Figure 1, the specific parent and peer context variables assessed or discussed in the papers found in this Special Issue were a balance of positive and negative constructs, whereas child and adolescent behavior/adjustment variables tended to focus on different adjustment difficulties, such as externalizing and internalizing problems. For the most part, the parent/peer constructs examined (e.g., parental monitoring and peer victimization) are social-emotional variables that have been studied in the past, consistent with the constructs highlighted in Ladd and Parke’s review [9]. One exception was Assari et al.’s paper [1] that focuses on cortical development, representing an emergent trend in developmental and family science to examine the neurological correlates of children and adolescent relationships [10]. Although the peer context is not examined in Assari et al.’s study [1], the conceptual model guiding the authors’ thought—that children’s brains and hence reading development are impaired by the consequences of parents’ low levels of education—has clear implications for children’s adjustment in the peer group, given the empirical links among poor brain development, executive function, academic performance, and peer relation problems [11].

**Theoretical trends**. The conceptual models used by the authors in this Special Issue build on an amazing array of existing theories and models. Some of the papers are guided by grand theories such as Attachment Theory (Cox et al. [3]; Hu et al. [5]), Social Learning Theory (Jespersen et al. [2]; Sigal et al. [7]), Ecological Theory (Hu et al. [5]), Behaviorism (Jespersen et al. [2]), and Self-Determination Theory (Hu et al. [5]). As in the extant literature, there really is no “grand theory” of child and adolescent socialization that includes both parent and peer contexts in this Special Issue. Instead, research in this field tends to be guided by mid-range theories, models, or frameworks that either combine existing theories or use parts of a larger theory, oftentimes to address or suggest “next steps” in a research area or for practice [12,13]. 

Existing mid-range models that guide the papers in this Special Issue include the Marginalization and Diminished Returns (MDR) Framework (Assari et al. [1]); Coercion Theory (Cox et al. [3]); a Risk and Protective Factor Framework (Havewala et al. [4]); a Diathesis-Stress Model of Anxious Solitude (Gazelle & Cui [8]); Gateway Theory, Self-Derogation Theory, and Primary Socialization Theory of Substance Use/Abuse (Sigal et al. [7]); multiple models of emotion socialization and regulation, including Meta-Emotion Theory, the Tripartite Model of Emotion Regulation, Eisenberg’s Emotion Socialization Model, and Gottman’s Meta-Emotion Philosophy and Typology of Emotion Socialization (Jespersen et al. [2]); and the Extended Process Model (Lindsey [6]).

In addition to highlighting these mid-range models, this Special Issue includes two papers that propose new conceptual models. The Cox et al. [3] and Jespersen et al. [2] papers both integrate and build upon multiple existing theories within a subarea area of child or adolescent development: immigrant adolescent behavior problems and emotion socialization, respectively. Cox et al.’s [3] model proposes that adolescents become vulnerable to deviant peer influence when their language becomes disparate from their parents’ and when conflict and alienation ensue. Jespersen et al. [2] postulate that children’s emotion-socialization experience is a function of the children’s activity, emotions, and their parents’ or peers’ responsivity styles. Each paper represents an important trend by suggesting a specific and complex interplay of parent and peer contexts on socioemotional development.

## 2. Findings: Additive, Multiplicative, and Transactional Mechanisms

The innovative approaches used by the researchers who contributed to this Special Issue examined one or more of four conceptual models involving families, peers, and child/adolescent adjustment (see Figure 1). The first model depicts the *independent (main) or additive effects* of the parent or peer context to child/adolescent adjustment. Multiple studies in this Special Issue (Gazelle & Cui [8]; Havewala et al. [4]; Lindsey [6]; Sigal et al. [7]) presented evidence that parenting and peer relationships/characteristics were significantly and additively related to child/adolescent adjustment when examined simultaneously, suggesting that relationships with mothers/fathers and friends can serve as unique contexts for socialization. The second model focuses on *mediation effects*, in which potential underlying mechanisms linking parenting and peer relationships to child and adolescent outcomes are explored. These types of models are critical in research as they elucidate various mechanisms or processes related to child/adolescent adjustment (see Sigal et al. [7]) and therefore can inform interventions focusing on at-risk youth [14]. Evidence for the *moderating effects* also were tested in our Special Issue. Moderators provide information regarding when or under what conditions the independent and dependent variables are related [15]. For example, the Special Issue results demonstrated that positive mother and father parenting moderated (i.e., attenuated) the link between peer and adolescent adjustment difficulties (see Havewala et al. [4]), showing that positive parenting can serve as a protective factor for at-risk youth. In addition, evidence in this Special Issue demonstrates that the associations among parents, peers, and child/adolescent adjustment are moderated by child ethnicity, demonstrating that the specific linkages between parents, peers, and child/adolescent adjustment may vary by ethnic background. Finally, a fourth model that was investigated in this Special Issue (see Gazelle & Cui [8]; Hu et al. [5]) focused on *transactional effects*, which reflect bidirectional associations between individuals and social contexts over time [16]. For example, the findings demonstrated that parenting, peer relationships, and adolescent adjustment (i.e., anxious solitude) were reciprocally related in longitudinal cross-lagged associations (Gazelle & Cui [8]). These results suggest that, in addition to being shaped by personal attributes, adolescents’ interpersonal relationships inside and outside of the family are mutually influential; experiences in one relationship shape the experiences in the other relationship.

## 3. Future Directions in Parent/Peer Influence Research

One suggestion for future research is the development of grand socialization theories that encapsulate experiences in relationships with parents and peers. In addition, given that children’s relationships with parents tend to be vertical (i.e., parent exerts greater control) whereas interactions with friends are more horizontal and balanced [17], future research would benefit from examining more specifically how these distinct relationship attributes translate into unique socialization experiences (i.e., additive effects) and perhaps link to unique child outcomes. Indeed, evidence in the literature has demonstrated that, while parenting and peer relationships are significantly and additively related to adolescent antisocial behavior, peer relationships may be more critical in the development of social skills [18]. It would also be advantageous for future work to capture the heterogeneity in the interactions children have within their relationships and how this may influence the socialization process. For example, in the context of play, parent–child relationships may be more horizontal and balanced (rather than vertical and hierarchical) than in other contexts [19]. Another suggestion centers on the interaction between genetic and environmental factors. Specifically, with a growing body of evidence in the literature showing that the impact of parenting and peer relationships on child and adolescent adjustment may be moderated by genetic characteristics [20,21], one avenue for future research is to explore whether the additive effects of parents and peers varies by genetic allele. Likewise, it would be particularly informative to investigate whether the specific mediation pathways linking parenting, peer relationships, and child outcomes differ by genetic allele. Finally, given the recent interest in trauma and resilience, researchers might do well to examine the inter-related role of parents and peer recent in children’s short and long reactions to adverse childhood experiences (ACEs). The ACEs typically examined are family based (as in the original study [22]), but there are clearly peer-based traumas such as bullying and ostracism, and researchers are just beginning to look at those in conjunction with family ACEs [23,24]. However, family and peer relationships also might serve a mediation or moderating role, in other words, serve as “protective and compensatory experiences” (PACEs; [25]) to mitigate risk and to increase the chances of resilience. 

Our hope is that this issue will inspire creative new approaches to developmental research, practice, and policy as the complexity of family and peer systems functioning together is embraced.
